# Effectiveness of nurses’ training about mechanical ventilation weaning on neonatal outcomes

**DOI:** 10.1186/s12912-025-03257-9

**Published:** 2025-06-17

**Authors:** Mohammed F. Alharbi, Salwa A. Marzouk, Nojoud Alrashidi, Mohammed H. Abu-Alghayth, Eman A. Mater, Marwa A. Ibrahim, Huwida Hamdy Abdelmonem, Hanan A. Mohammed, Shadia A. Syan, Aml S. Abdelrahem, Ahlam D. Alshehri, Shimaa M. Moursy, Abeer A. Almowafy, Faransa A. Ahmed

**Affiliations:** 1 Department of Nursing Management and Education, College of Nursing, Taif University, Taif, 2425 Saudi Arabia; 2 College of Nursing, University of Hail, Hail, Saudi Arabia; 3 Department of Medical Laboratory Sciences, College of Applied Medical Sciences, University of Bisha, P.O. Box 255, Bisha, 67714 Saudi Arabia; 4 College of Nursing, Taibah University, Taibah, Saudi Arabia; 5 Faculty of Nursing, Cairo University, Cairo, Egypt; 6 Faculty of Nursing, Galala University, Suez, Egypt; 7 Faculty of Nursing, Fayoum University, Fayoum, Egypt; 8 Faculty of Nursing, Sohag University, Sohag, Egypt; 9 College of Applied Medical Sciences, King Faisal University, Hofuf, Saudi Arabia; 10 Faculty of Nursing, Minia University, Minia, Egypt; 11 College of Applied Medical Sciences in Alnamas, University of Bisha, Alnamas, Saudi Arabia; 12 Faculty of Nursing, Assiut University, Assiut, Egypt; 13 International Islamic Institute for Population Studies and Research, Al-Azhar University, Cairo, Egypt

**Keywords:** Nurses, Training, Neonates, Mechanical ventilation weaning

## Abstract

**Introduction:**

Prematurity is a significant global health challenge. Premature infants frequently need invasive mechanical ventilation until their lungs are fully developed. Due to the possible complications of ventilation, nurses in the neonatal intensive care unit (NICU) must deliver specialized care to achieve the best outcomes for these infants.

**Objective:**

This study aimed to explore the effectiveness of nurses’ training in mechanical ventilation weaning on neonatal outcomes.

**Method:**

A quasi-experimental non-equivalent group design was used with purposive sampling of 70 nurses and 64 newborn infants on invasive mechanical ventilation. The infants were divided into two groups: 32 weaned by trained nurses (study group) and 32 weaned by standard methods (control group). Data was collected using a structured questionnaire about the nurses and neonates. A well-designed training program, including theoretical and practical components, was conducted for the nurses to ensure proper weaning of neonates from mechanical ventilation.

**Results:**

The study group demonstrated a significant reduction in the use of surfactant replacement therapy post-extubation compared to the control group (*p* = 0.003). Additionally, infants in the study group experienced a statistically significant decrease in NICU hospitalization duration, total weaning time, and total ventilation period compared to the control group (*p* = 0.003, 0.0001, and 0.0001, respectively). Complications were markedly lower in the study group, with two-thirds of infants experiencing no complications, compared to 15.6% in the control group (*p* = 0.001). Moreover, re-intubation rates were significantly reduced in the study group compared to the control group (*p* = 0.1026).

**Conclusion:**

These results highlight the effectiveness of the intervention in improving clinical outcomes for neonates, including reduced treatment needs, shorter hospital stays, and fewer complications.

**Clinical trial number:**

Not applicable.

**Supplementary Information:**

The online version contains supplementary material available at 10.1186/s12912-025-03257-9.

## Introduction

Premature birth remains a significant global health issue, which represents a major cause of neonatal morbidity and mortality in both developed and developing countries. Around 15 million infants are born preterm each year worldwide [1]. A large proportion of these births, approximately 85%, occur in developing nations [2]. In Egypt, the preterm birth rate is estimated to be below 10% in the general population [3].

Premature infants face unique challenges due to their underdeveloped organs and immune systems; they are highly susceptible to breathing and feeding difficulties, growth delays, temperature regulation problems, and infections [4].

One of the most serious complications associated with prematurity is respiratory distress syndrome (RDS), a common cause of breathing difficulty in newborns. Despite advancements in treatment options (such as antenatal corticosteroids, surfactants, and advanced neonatal respiratory support), RDS remains a leading cause of illness and death among preterm infants [5].

Mechanical ventilation is a vital treatment for newborns with respiratory distress, supporting essential gas exchange. While non-invasive ventilator support for neonates has advanced, invasive ventilation through an endotracheal tube remains a standard practice in NICUs [6].

## Review of literature

Previous research reported that the nurses who receive proper training regarding mechanical ventilation are better able to identify when newborns are ready to be weaned, perform correct weaning techniques, and monitor for any potential complications [7].

Qualified nursing staff is critical to reducing the time patients spend on mechanical ventilation and the associated risks, such as ventilator-associated pneumonia and lung injury [8].

Furthermore, enhancing nurses’ competencies through targeted education can lead to improved clinical practices and better patient outcomes [9].

Ventilation serves as a temporary support to assist until an infant’s lungs can function independently. However, extended use can lead to several complications, such as atelectasis, post-extubation stridor, perioral tissue damage, ventilator-associated pneumonia, mucus plugging, pneumothorax, pneumomediastinum, and ICU neuromyopathy [10]. Therefore, stopping mechanical support as quickly as possible should be a top focus for critical care nurses [11].

Weaning from ventilation is the gradual process of reducing external respiratory support to encourage independent breathing. This process is complex in preterm infants as their lungs are still developing, so it must be carefully managed to minimize stress on their respiratory system while avoiding a premature return to ventilation [6].

One of the causes of extubation failure is inadequate training of medical personnel, so specialized neonatal nursing care is central to optimizing the care of such critical newborns [12].

Nurses play a vital role in the weaning process by assessing infants’ readiness for weaning from mechanical ventilation, adjusting ventilation settings, and implementing infection control measures. Their role can be enhanced with training in early weaning protocols, respiratory distress monitoring, and effective neonatal team communication [13]. The findings of this study are particularly important as they enhance nurses’ knowledge and improve their clinical practice related to the care of neonates during the weaning process from mechanical ventilation, ultimately benefiting neonatal outcomes. Therefore, nurses need to develop these skills during their clinical practice. So, this study aimed to explore the effectiveness of nurses’ training in mechanical ventilation weaning on neonatal outcomes. This result was achieved by developing and implementing a training program for nurses regarding mechanical weaning and investigating the effect of this intervention (nursing training) on neonatal outcomes.

### Research questions

How effective is the training program for nurses in mechanical ventilation weaning on improving neonatal outcomes?

### Null hypothesis (H₀)

The implementation of a training program for nurses on mechanical ventilation weaning has no significant effect on neonatal outcomes.

### Alternative hypothesis (H₁)

Neonates cared for by nurses who have undergone the training program will exhibit better outcomes from mechanical ventilation weaning than those cared for by untrained nurses.

## Methods

### Study design

A quasi-experimental, non-equivalent control group design was employed through the period from April to September 2023 to evaluate the impact of nurse training on mechanical ventilation weaning practices and subsequent neonatal outcomes.

### Study setting

The study was conducted in the NICU at Assiut Children’s University Hospitals, Assiut, Egypt, and the NICU at Al-Namas General Hospital, KSA.

#### Assiut university children hospital

This unit services more than one province, from El-Minia to Red Sea province. It contains 50 incubators, with the mean of total admission every month being 140 neonates. It includes an examination room, feeding preparation, clinical pharmacy, incubator sterilization, breast feeding room, and eight rooms for caring for critically ill neonates.

#### Al-Namas general hospital

affiliated with the Ministry of Health. It involves 42 incubators, with the mean total admission every month being 80 neonates; it includes an examination room, feeding preparation, clinical pharmacy, incubator sterilization, septic room, and big hall for caring for clinically stable neonates and two rooms for caring for critically ill neonates.

### Study population and sampling

A total of 70 registered nurses from selected NICUs were recruited through convenience sampling. Inclusion criteria include nurses with at least six months of experience in a NICU setting. Exclusion criteria involved nurses who are not directly involved in the management of mechanical ventilation. The researchers contacted appropriate nurses in the hospital’s NICU with information about the study. The written consent was obtained from volunteers who completed the questionnaire.

Regarding newborn infants due to the limited sample size, the researchers use the census method. Within two months before the intervention (control group) and two weeks after the intervention (study group), all infants admitted to the NICU of eligible neonates were chosen for the study. A purposive sample from nurses was obtained due to constraints in a clinical setting. This approach ensured a comprehensive evaluation of the training intervention’s impact on all eligible neonates within the specified timeframes.

### Inclusion/Exclusion criteria

Inclusion criteria: Involved newborns are ≥ 32 weeks of gestational age (according to maternal last menstrual period, or the Ballard scores), delivering by vaginal or cesarean section delivery, hospitalized in the NICU and being connected to a ventilator for more than 24 h, while newborns with congenital anomalies and severe asphyxia are excluded.

### Study tools

A structured questionnaire was employed to collect data relevant to the study. The researchers administered this questionnaire, which comprised the following data:

- **Nurses’ Profile.** This section collected data on the personal and professional characteristics of the nurses, including gender, age, marital status, educational background, NICU work experience, employment status, and details of any relevant training courses completed.
- **Baseline Knowledge of Nurses about Mechanical Ventilation techniques and protocols**, including assessment of oxygen saturation, measuring blood pressure of neonates, success points for suctioning procedures, and precautions during weaning ventilation.
- **Demographic Characteristics of the Neonates.** Data were extracted from the infants’ medical records, including gender, age, weight, and mode of delivery.
- **Medical History of the Neonates.** This section documented the neonates’ date of admission, medical diagnosis, date of intubation for mechanical ventilation, time to extubating, age at weaning initiation, and discharge date. The length of hospital stay was calculated from the date of admission to discharge.
- **Cardiorespiratory Assessment.** Key physiological parameters such as heart rate, respiratory rate, and oxygen saturation levels were continuously monitored over 24 h.
- **Assessment of surfactant replacement therapy and incidence of ventilator-related complications.** Major complications include atelectasis, pneumonia, bronchitis, and fever, while minor complications include nasal injury, gastroesophageal reflux, pressure sores, skin breakdown from ventilation equipment, and sinusitis.

#### Procedure

Official approval was secured from the Dean of the Faculty of Nursing and key hospital authorities before initiating the study. After finalizing the study tools, the program was completed, data collection and analysis were conducted over three months, from June to August 2023.

- **Training Planning**: The training program was designed based on an initial assessment of the nurses’ baseline knowledge, which informed the selection of relevant training topics. A total of 70 nurses participated in the program. The researchers explained the study’s objectives to the participants to ensure cooperation, and written consent was obtained from all participants.
- **Training implementation**: the researchers present three days per week from 9 AM to 1 PM at the study site for two weeks to implement two sessions as the theoretical component of the training and two sessions to cover the practical part of care for high-risk infants undergoing extubation mechanical ventilation. For the application, each session took 30–40 min, and the theory or presentation sessions and re-clarification sessions were conducted according to nurses’ readiness. After completing the period of training, 32 neonates were weaned by trained nurses, while standardized methods managed the other 32.
- **Training evaluation**: To assess the program’s effectiveness, neonatal criteria were measured (before and after the intervention), such as the presence of secretion, color of sputum, surfactant replacement therapy, oxygen saturation, and respiratory rate. In addition to measurement of neonatal outcomes after the nurses have implemented the training, including improvement in respiratory function, presence of complications, total weaning period, total ventilation period, reintubation, and length of hospital stay.

#### Program description

The training program included developing structured nursing training for high-risk neonates on mechanical ventilation. Key components covered included:

- **Theoretical component**: it includes raising awareness about the weaning preparation protocols, indications for extubating, post-extubating care, nursing interventions during and after extubating, as well as the identification of warning signs and common complications that may arise post-extubating. Researchers formulated these components following a comprehensive review of relevant literature, including nursing textbooks, peer-reviewed journals, and credible online resources.
- **Practical component**: it encompassed essential skills such as hand hygiene, vital sign assessment, respiratory hygiene (including endotracheal tube (ETT) care, oral and nasal suctioning), monitoring sputum (type and amount), care during endotracheal tube removal, and capillary and venous blood sampling. Moreover, how to keep documentation for any intervention or changes.
- **Program training tools**: The researchers employed a variety of teaching methodologies, including lectures, group discussions, demonstrations, and re-demonstrations, supplemented by a guiding booklet with colored illustrations, video presentations, and PowerPoint slides presented on the researchers’ laptops.
- **Supportive materials**: Ongoing access to resources, such as online modules, reference guides, and access to clinical experts for additional support post-training were provided.

### Pilot study

The researcher involves 6 infants at first to assess the effectiveness, comprehensibility, and feasibility of the training program for nurses with ensuring the validity and reliability of data collection instruments.

### Ethical considerations

The study received ethical approval from Assuit University with the code number (IRB No: 1120230609). Researchers introduced themselves to parents of eligible newborns, explained the study’s purpose, and obtained informed written consent. Data confidentiality was ensured through encryption and the use of unique code numbers. NICU nurses were also informed about the study, and written consent was obtained from those who volunteered to participate and complete the questionnaire. Informed consent was obtained from all participants in the research, and their participation was entirely voluntary and not-for-profit. All participants gave informed consent for the research, and their anonymity was preserved after explaining the purpose of the study and guaranteeing data confidentiality.

### Statistical analysis

After the necessary data were collected and verified, encoding, verification, and analysis were conducted using IBM SPSS for Windows software version 25 [14]. The normality of the data was tested using the Kolmogorov-Smirnov and Shapiro-Wilk tests [15]. Descriptive statistics were utilized to summarize the data, presenting quantitative data as mean ± standard deviation and nominal and ordinal data as frequencies and proportions. The Chi-square test was applied to compare qualitative data between groups, while the independent sample t-test was used to compare quantitative data. Statistical significance for all tests was set at a p-value of 0.05.

## Results

The study included 70 nurses who worked in the NICU and received the intervention program. About two-thirds (62.7%) of nurses were less than 30 years and 59.3% graduated from secondary school of nursing. More than two-fifths of them (44.6%) had experience from 10 to 15 years, and the vast majority (98.3%) of them did not attend training courses about neonatal caring on mechanical ventilation.

**Table 1:** General characteristics of the studied infants in both groups study and controls

Item	Study (*n* = 32)		Control (*n* = 32)		*P*-value
	NO.	%	NO.	%	
**Sex**:					0.965626
Male	18	56.3	17	53.1	
Female	14	43.7	15	46.9	
**Mode of delivery**:					0.651276 0.968883
NVD	14	43.7	12	34.5	
C.S	18	56.3	20	62.5	
**Gestational age**: (weeks)					
< 33	7	21.9	9	28.1	
34–37	19	59.3	16	50	
38–41	4	12.5	4	12.5	
≥ 42	2	6.3	3	9.4	
**Birth weight in grams at admission**: Mean ± SD	2200.50 ± 0.79		2200.65 ± 0.76		0.4418
**Age at the beginning of the weaning process (days)**: Mean ± SD	9.45 ± 2.6		10.18 ± 14.17		0.7753
**Medical diagnosis**					
Respiratory distress syndrome	10	31.2	10	31.2	0.9993
Neonatal sepsis	7	21.9	6	18.8	
Bronchopneumonia	7	21.9	8	25	
Transient tachypnea of the NB	8	25	8	25	

Table (1) shows the characteristics of the studied neonates in the study and control groups. The higher percent of the study and control groups were male (56.3% and 53.1%, respectively), and the mean weight was 2200.50 ± 0.79 g for the study group and 2200.65 ± 0.76 g for the control group. Regarding infant age at the beginning of the weaning process, the mean age of infants in the study group was 9.45 ± 2.6 days, while 10.18 ± 14.17 days old for the control group. In addition, the higher percent of neonates in both groups were preterm with gestational age 34–37 weeks and were diagnosed with respiratory distress syndrome. All differences were not statistically significant.

**Table 2:** Comparison of infants’ cardiorespiratory function between the two groups before and after extubation

Item	Before extubation				*P*-value	After extubation				*P*-value
	Study (32)		Control (32)			Study (32)		Control (32)		
	NO.	%	NO.	%		NO.	%	NO.	%	
**Presences of secretion**	11	34.4	20	62.5	0.000112	5	15.6	14	43.75	0.000112
**Sputum color**										
White	13	40 0.6	12	37.5	0.957136	15	46.9	7	21.9	0.003322
Green	9	28.1	9	28.1		9	28.1	11	34.4	
Purulent	10	31.3	11	34.4		8	25	14	43.7	
**Surfactant replacement therapy**	13	40.6	15	46.9	0.786649	7	21.9	13	40.6	0.003322
**Respiration rate** (Mean ± SD)	39 ± 7.16		45.55 ± 7.23		0.0006	35 ± 8.12		40.32 ± 7.11		0.0070
**O2 saturation** (Mean ± SD)	96.82 ± 2.80		92.89 ± 1.50		0.0001	99.90 ± 2.86		95.90 ± 1.64		0.0001

The comparison of infants’ cardiorespiratory function between the two groups before and after extubation is in Table (2). However, there was a statistically significant decrease in the presence of secretion and a statistically significant improvement in respiratory function and O_2_ saturation in the study group compared to the control group either before or after extubation. There was a considerable decline in the use of surfactant replacement therapy among the infants in the study group compared to control one after extubation (p value = 0.003322).

Table 3 presents that there was a statistically significant decrease in the duration of hospitalization in NICU, total weaning, and total ventilating period among infants in the study group compared to the control group (*P* = 0.0035, 0.0001 & 0.0001, respectively). Regarding complications, about two-thirds of infants in the study group had no complications compared to 15.6% of the control group, with a statistically significant difference (*P* = 0.0011). Additionally, there was a statistically significant reduction regarding re-intubation in the study group compared to the control groups(*P* = 0.1026).

**Table 3:** Comparison between both groups regarding neonatal outcomes after extubation

Item	Study (*n* = 32)		Control (*n* = 32)			*P*-value
	NO.	%	NO.	%		
**Total hospital stay (days)**						0.0035
Mean ± SD	20.45 ± 2.6		28.18 ± 14.17			
**Total weaning period (days)**						0.0001
Mean ± SD	4.91 ± 1.97		7.22 ± 1.52			
**Total ventilation period (days)**						0.0001
Mean ± SD	7.59 ± 0.72		12.94 ± 0.78			
**Complications**						
No complications	11	34.4	5	15.6		0.001096
Minor complications	10	31.2	7	21.9		
Major complications	11	34.4	20	62.5		
**Re-intubation**	3	9.4	8	25		0.010264

**Fig. 1:**
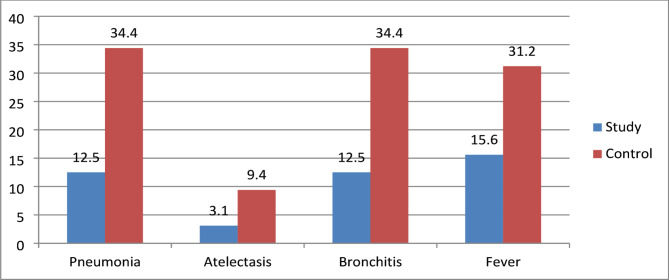
Comparison between the study and control group regarding major post-extubation complications

Figure (1) illustrates that there was a statistically significant reduction in major post-extubation complications such as pneumonia, atelectasis, bronchitis, and fever in the study group compared to the control group.

## Discussion

Mechanical ventilation provides life-support breathing assistance for premature infants who cannot breathe independently. Therefore, delivering proper nursing care to neonates on mechanical ventilation is essential for effective management, successful weaning, and positive outcomes [16]. Accordingly, this study was conducted to examine the impact of a training program for NICU nurses on mechanical ventilation weaning and its effects on neonatal outcomes in the form of ventilator-related complications.

The study involved 64 premature babies and 70 NICU nurses who participated in an intervention program. Most nurses were under 30 years old, around half of them were graduates of secondary nursing schools, and had 10–15 years of experience, while the vast majority had not attended training on neonatal mechanical ventilation.

These findings are similar to the findings of the previous studies [17, 18]. Most intervention programs are directed to less experienced and moderately skilled nurses in the NICU who lack specialized training, as managing mechanical ventilation in newborns demands specific expertise due to the fragility of infants’ respiratory systems. Without adequate training, nurses may feel less prepared or confident in handling these situations, which could affect the quality of care and heighten the risk of complications.

Regarding the characteristics of the studied infants in the study and control groups, the results of the current study showed similarities between the two groups in terms of sex, mode of delivery, birth weight, medical diagnosis, and mean gestational age at admission. The balanced characteristics between groups strengthen the validity of the study, allowing for a focused analysis of the intervention’s effects without major confounding influences from baseline differences.

Regarding the impact of the training program on cardiorespiratory function, the findings of this study indicate a significant statistical difference in cardiorespiratory outcomes, specifically in secretion presence and O₂ saturation, both before and after extubation when comparing the study and control groups. These results suggest that the training program can enhance nurses’ knowledge and skills, enabling them to deliver high-quality care to infants and improve infant outcomes. Our findings align with a previous study [19] on training nurses in weaning premature infants from mechanical ventilation, conducted on 312 preterm infants in a Tehran NICU. That study reported a notable reduction in secretion presence and improved O₂ saturation in the post-training phase. Also, the results of a previous study [5] in India demonstrated a significant improvement in the nurses’ skill in handling ventilator weaning, resulting in fewer respiratory complications and increased oxygen saturation among infants.

Moreover, current results have shown that the intervention applied to the study group had a clear positive impact on several key outcomes for infants, including a reduction in NICU stay, faster weaning, shorter ventilation periods, and fewer complications compared to the control group.

Earlier research was confirmed, and the impact of a nursing care training program on the outcomes of mechanically ventilated infants in Tehran was examined. The study, involving 120 ventilated infants, showed a significant improvement in neonatal outcomes, with a statistically significant reduction in most post-extubation complications following the intervention [17]. Another study [20] in Iran assessed the effect of a comprehensive nursing care protocol on preterm, mechanically ventilated infants. They reported significant reductions in respiratory complications such as bronchopulmonary dysplasia (BPD) and ventilator-associated pneumonia. Additionally, re-intubation rates decreased, which led to shorter ventilation duration and fewer overall complications. The reductions in NICU hospitalization duration, weaning period, and total ventilation time are likely linked to the increased competence and attentiveness of trained nurses, which have been documented in other research to lead to faster stabilization and recovery in infants [21, 22].

Finally, this study can serve as a foundation for future, more in-depth research on training programs for nurses related to weaning from mechanical ventilation and its impact on neonatal outcomes. Additionally, significant efforts were made to standardize the program, focusing on enhancing nurses’ understanding of neonatal care during mechanical ventilation and building the skills needed to address associated challenges.

### Strength and limitations

Finally, the study focuses on the training of nurses in the weaning process of artificial ventilation, highlighting a vital component of newborn care. This emphasis helps evaluate the training’s impact on patient outcomes. The results may inspire similar training programs to improve newborn care worldwide. Utilization of Quantifiable statistics like post-extubation complication rates and reintubation rates enhances the validity of training intervention evaluations. The presence of a control group improves the study’s ability to determine the intervention’s causal effect on neonatal outcomes. Documenting statistically significant findings enhances the credibility of the results, indicating that the observed gains are unlikely to be attributable to chance.

Due to the absence of randomization, the study is susceptible to potential selection bias. The groups may differ in ways that could influence the outcomes, such as baseline health disparities among neonates or variations in staffing levels and expertise across different units. Additionally, the lack of a post-training knowledge assessment for nurses presents a limitation in evaluating the effectiveness of the training program in enhancing their knowledge and skills.

### Implications for practice

To optimize the care of neonates requiring invasive mechanical ventilation in NICUs, it is essential to provide nurses with regular, high-quality training. This study demonstrates the effectiveness of such training in improving nurses’ knowledge and skills. Given the initial skill deficits identified, ongoing training and research are necessary to ensure that nurses are equipped to provide optimal care for these vulnerable infants.

## Conclusion

The implementation of a training program for nurses on mechanical ventilation weaning significantly improved respiratory function and oxygen saturation in infants with a reduction in the total ventilation duration, incidence of complications, and length of hospital stay. These results underscore the positive impact of targeted nurse education on neonatal care outcomes.

Hence, future multicenter studies are necessary to substantiate further the effect of nurse training programs on ventilator withdrawal processes and their impact on neonatal health outcomes.

### Recommendation

In the light of the study findings, it is recommended that:

- Healthcare institutions must establish and execute structured training programs tailored for nursing staff, emphasizing evidence-based practices related to mechanical ventilation weaning protocols.
- Continuous education and professional development opportunities must be accessible for nurses in neonatal and pediatric units.
- Further research into the long-term effects of nurse-led training programs on mechanical ventilation outcomes.

## Electronic supplementary material

Below is the link to the electronic supplementary material.

Supplementary Material 1

## Data Availability

Data is provided within the manuscript or supplementary information files.
