# Supplementary material for: Effectiveness of nurses’ training about mechanical ventilation weaning on neonatal outcomes
Source: BMC Nurs. 2025 Jun 17;24:654. doi: 10.1186/s12912-025-03257-9 (PMC12172335; doi:10.1186/s12912-025-03257-9)
Supplement: Supplementary file 1 — Supplementary Material 1 [file 12912_2025_3257_MOESM1_ESM.pdf]

I) Astructures interview questioner about Effectiveness of Nurses' Training about Mechanical Ventilation Weaning on Neonatal Outcomes

Code No:
Date:

Nurses' Personal characteristics: -

1. **Name**: (optional)………………..

2. **Age**:

A- From 20 to less than 25 year

B- From 25 to less than 30 years

C- From 30 to less than 35 years old

D- 35 years or more

3. **Gender**:

A- Male B- Female

4. Educational Qualification:

A- Diploma of Nursing C- Bachelor of Nursing

B- Health Technical Institute D-Post Graduate Studies

5. Years of Experience in NICU:

A- Less than 5 years C- From 10 years to less than 15 years

B- From five years to less than 10 years D- 15 years or more

6. Training courses in nursing care for neonates undergoing mechanical ventilation

A- Yes B- No

II: Characteristics of Neonates

Chronological age:

1- Less than 24 hours. 2-From 1 to less than 10 days.

3-From 10 to less than 28 days. 4- 28 days or more.

Gestational age:

1-Less than 37 weeks

2-From 37 to 42 weeks

3-More than 42 weeks

Gender:

1-Male

2-Female

Birth weight:

1-Less than 1.500 Kg.

2-From 1.500 to less than 2.500 Kg.

3-From 2.500 to less than 3.500 Kg.

4- From 3.500 Kg or more.

Medical Diagnosis:

-………………….

Type of labor

1- Normal delivery
2- Cesarean section

- **Date of admission at NICU………………….**
- **date of intubation on MV………………**
- **times from extubation, age in the beginning of the weaning process……………………………………..**
- **post-extubation complications……………………………..**
  …………………………………………………

- **Length of hospital stay (……../days)**
- **Date of discharge………………………………**

cardiopulmonary assessment chart of newborn

Age……………..
Gender…………………
Diagnosis…………….
Birth weight………………………gm
Date of admission………………..
Date of discharge…………………

Assessment	/Hours	Remarks
Heart rate		
Respiratory rate		
O2 saturation		

Assessment of nurses' knowledge about the following: -

1-Blood oxygen saturation in newborns with persistent pulmonary hypertension ranging from:

A- From 85% to less than 90%

B- From 90% to less than 93%

C- From 93% to 95%

D- From 96% to 100%

2-When there is a sudden decrease in the percentage of oxygen on the monitor, the following should be done:

A- Inform the treating physician directly

B- Change ventilator parameter's settings

C- Reviewing the pulse ox meter in place then informing the treating physician

D- Done suctioning and re-heating of newborn

3. When measuring blood pressure for newborns with persistent pulmonary hypertension, it should be measured:

A- From the right arm only

B- From the left arm only

C- From the right arm and the right leg

D- From the right arm and the left leg

4. When dealing with a ventilator, the following should be done

A- You must review the device settings with the doctor's orders

B- Ensure that the humidifier is functioning with sterile water

C- You must make sure that the ventilator connections are intact

D- All of the above

5. Tools to replace the endotracheal tube for newborn:

A- Ampopag and Mask

B- Suction Catheter

C- Laryngoscope and endotracheal tube different sizes

D- All of the above

6. Which of the following points illustrates the success of the suctioning procedure from the endotracheal tube:

A- Improved oxygen saturation in tissues

B- Reduced exertion rate during breathing

C- Improved respiratory and heart rate

D- All of the above

7. One of the complications that occur during suctioning from the endotracheal tube:

A- Cardiac dysrhythmias

B- Occurrence cyanosis to the neonate

C- It is possible to move the endotracheal tube from its place

D- All of the above

8. When a child's cyanosis occurs during suctioning, what is the procedure to be taken?

A- Immediately stop suctioning

B- Connect it to oxygen

C- Inform the doctor

D- All of the above

9- Presences of secretion

No

Yes

10- Sputum color

White

Green

Purulent

11-Surfactant replacement therapy

No

Yes

In case of yes pleas fill the next sheet

III) Assessment of nurses' knowledge about surfactant replacement therapy in neonates

- **Indications for surfactant replacement**

…………………………………

……………………………………

- **Usual dose-------------------------**
- **Methods of administration………………………….**

- **Timing of administration…………………………..**

- **Adverse effect** …………………………………………
